# Estimated average blood glucose level based on fructosamine level

**DOI:** 10.20945/2359-3997000000589

**Published:** 2023-01-25

**Authors:** Luis Jesuino de Oliveira Andrade, Alcina Maria Vinhaes Bittencourt, Luiz Felipe Moreno de Brito, Luís Matos de Oliveira, Gabriela Correia Matos de Oliveira

**Affiliations:** 1 Universidade Estadual de Santa Cruz Departamento de Saúde Ilhéus BA Brasil Departamento de Saúde, Universidade Estadual de Santa Cruz, Ilhéus, BA, Brasil.; 2 Universidade Federal da Bahia Departamento de Medicina Salvador BA Brasil Departamento de Medicina, Universidade Federal da Bahia, Salvador, BA, Brasil.; 3 Laboratório de Análises e Pesquisas (LAP) Itabuna BA Brasil Laboratório de Análises e Pesquisas (LAP), Itabuna, BA, Brasil.; 4 GELL Clinic Itabuna BA Brasil GELL Clinic, Itabuna, BA, Brasil.; 5 Centro Universitário UniFTC Faculdade de Medicina Salvador BA Brasil Faculdade de Medicina, Centro Universitário UniFTC, Salvador, BA, Brasil.

**Keywords:** Fructosamine, glucose values, diabetes mellitus control

## Abstract

**Objective:**

To define the mathematical relationship between fructosamine levels and average glucose values.

**Subjects and methods:**

The study comprised laboratory data of 1,227 patients with type 1 or 2 diabetes mellitus. Fructosamine levels measured at the end of a 3-week period were compared against the average blood glucose levels of the previous 3 weeks. Average glucose levels were determined by the weighted average of the daily fasting capillary glucose results performed during the study period, and the plasma glucose measured in the same sample collected for fructosamine measurement.

**Results:**

In total, 9,450 glucose measurements were performed. Linear regression analysis between fructosamine levels and average glucose levels showed that for each 1.0 µmol/L increase in fructosamine level there was a 0.5 mg/dL increase in average glucose level, as estimated by the equation *Mean glucose level = (0.5157 x Fructosamine) – 20.* The coefficient of determination (r^2^ = 0.353492, p < 0.006881) allowed the calculation of the estimated average glucose based on fructosamine level.

**Conclusion:**

Our study demonstrated a linear correlation between fructosamine level and mean blood glucose level, suggesting that fructosamine levels can be a proxy for the average glucose level in assessing the metabolic control of patients with diabetes.

## INTRODUCTION

Fructosamine (1-amino-1-deoxy-d-fructose) is a term used for all glycated plasma proteins, particularly albumin (which represents 55%-80% of all serum glycated proteins), immunoglobulins, and various proteins. Fructoses are ketoamines formed by irreversible non-enzymatic combination of glucose and plasma proteins via glycation. The glycation process corresponds to the initial formation of a Schiff base (aldimine) that is subsequently rearranged into a stable Amadori product (ketoamine) as a function of the binding of the amino acids cysteine, arginine, and lysine to glucose ( 1 ).

Fructosamine values reflect the blood glucose levels over a period of 2-3 weeks; thus, fructosamine constitutes an alternative biomarker of glycemic control when glycated hemoglobin (HbA1c) is not indicated for this purpose ( 2 ). The fact that fructosamine reflects the average glucose over a 2-3-week period allows for more accurate glycemic control and better therapeutic adjustment, especially in patients with unstable diabetes mellitus (DM) and is, thus, more suitable for monitoring of treatment response.

Fructosamine has not been used as much as HbA1c for monitoring metabolic control in DM, although several studies indicate that fructosamine may be superior to HbA1c in this setting ( 3 ). As an added advantage, fructosamine levels are not affected by hemoglobinopathies (structural hemoglobin variants and thalassemia syndromes) or anemias, like HbA1c levels are, and may be used in circumstances in which HbA1c measurement is unreliable due to biological or analytical interferences ( 4 ). A study comparing fructosamine versus HbA1c measurements has shown both markers to have a good correlation in patients with DM but not in normal subjects ( 5 ).

Some time ago, fructosamine measurement was validated as a standard for short-term glycemic control in patients with DM ( 6 ). Based on these considerations, the aim of this study was to establish a mathematical relationship between serum fructosamine levels and average blood glucose values.

## SUBJECTS AND METHODS

The study included laboratory data collected from 1,227 patients with DM, including 61 with type 1 DM and 1,155 with type 2 DM. Serum fructosamine levels obtained and measured at the end of a period of 3 weeks were compared with plasma glucose levels and average glucose levels obtained in the previous 3 weeks. Mean glucose levels were estimated as the mean value of daily capillary glucose measured at fasting during the study period and the glucose measured in the same sample collected for measurement of fructosamine. Considering that capillary blood glucose levels are approximately 2-5 mg/dL higher than venous plasma glucose levels in the fasting state, we decreased 3.5 mg/dL (mean 2-5 mg/dL) from the weighted average of the daily capillary glucose results for the statistical analysis.

Capillary glucose was determined using the glucose oxidase method with glucometers of various brands. Serum glucose levels were measured using a glucose analyzer with a coefficient of variation of 2%, while serum fructosamine levels were measured using colorimetric assay, with reference values of 205-285 μmol/L and a coefficient of variation of 3.94%. All serum fructosamine and plasma glucose tests were measured in the same laboratory, while capillary blood glucose tests were self-assessed.

The study was conducted at the outpatient level, and none of the participants had changes in laboratory parameters that could interfere with the fructosamine measurements, including changes in hemoglobin, bilirubin, ascorbic acid, or serum albumin concentrations.

### Statistical analysis

The statistical analysis was performed using Excel (Microsoft, Seattle, WA, USA) for assessment of the correlation between serum fructosamine levels and mean glucose levels. Spearman's correlation was used to assess the association between fructosamine and mean capillary blood glucose, and linear regression was applied to evaluate the mathematical relationship between fructosamine levels and fasting plasma glucose. P levels < 0.05 were considered statistically significant across all calculations.

According to Resolution CNS 510/2016, our study was not required to be approved by the ethics committee due to the fact that the research aimed at deepening the theoretical understanding of a situation that arises spontaneously and contingently in professional practice and did not reveal data that could identify the research subjects.

## RESULTS

A total of 9,450 glucose and 1,227 fructosamine measurements were performed. Linear regression analysis between fructosamine and mean glucose level ( Table 1 ) demonstrated that for every 1.0 μmol/L rise in fructosamine level there was a 0.5 mg/dL rise in mean glucose level, as estimated by the following equation:

Mean glucose level = (0.5157 x Fructosamine) – 20

**Table 1:** Fructosamine levels and corresponding estimated mean glucose levels calculated using the formula mean glucose level = (0.5157 x fructosamine level) – 20

Fructosamine level µmol/L	Estimated mean glucose level mg/dL *
205	85.7
215	90.8
225	96.0
235	101.1
245	106.3
255	111.5
265	116.6
275	121.8
285	126.9

* Average glucose levels = 0.5157 x Fructosamine - 20.

The coefficient of determination (r^2^ = 0.353492, *p* < 0.006881) ( Table 2 ) indicated that it was possible to calculate the estimated average glucose based on fructosamine level ( Table 3 ).

**Table 2:** Coefficient of determination

Regression Statistics	
Multiple R	0.594552
R squared (r^2^)	0.353492
Adjusted R squared	0.352045
Standard error	49.30802
Observations	1227

**Table 3:** Intersection between fructosamine and blood glucose

	Coefficient	Standard error	Stat t	P value
Intersection	23.28142	8.574671	2.715138	**0.006881**
Fructosamine	0.404109	0.025849	15.6335	2.91E-44

**Figure 1:**
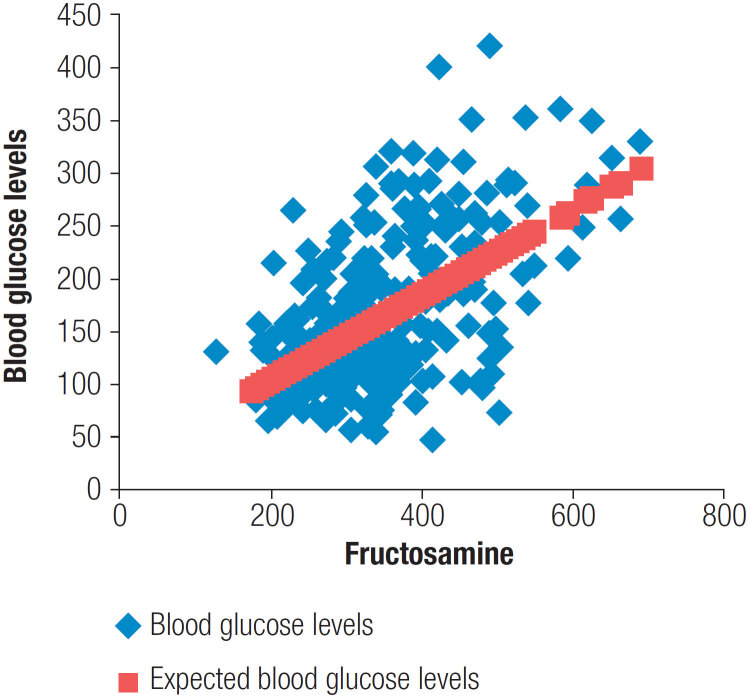
Relationship between mean glucose levels and fructosamine levels.

Figure 1 shows the relationship between mean glucose levels and fructosamine levels.

## DISCUSSION

In this study, we established a mathematical relationship between fructosamine levels and mean glucose levels following the equation *Mean glucose level = (0.5157 x Fructosamine) – 20.*

Human albumin is an extracellular multifunctional protein that has recently become a biomarker of hyperglycemia. Fructosamine is a reliable biomarker of glycemic control that has low cost and easy laboratory evaluation, showing good correlation with the mean plasma glucose over a period of 2-3 weeks. The term fructosamine is attributed to all ketoamine linkages that result from glycation of serum proteins ( 2 ). Unlike fructosamine, which provides short-term information on prior glycemic control (2-3 weeks prior), HbA1c provides long-term information about the previous average glycemic control (2-3 months) ( 7 ). However, the rate of non-enzymatic glycation of fructosamine is about 9-10 times higher than that of HbA1c.

The criteria for diagnosis of DM, according to the American Diabetes Association, include measurement of HbA1c level or plasma glucose level (fasting plasma glucose or the 2-hour plasma glucose value after a 75-g oral glucose tolerance test) ( 8 ).

Another study evaluating the mathematical relationship between HbA1c and average blood glucose levels over the previous 3 months in patients with DM has proposed the following equation to estimate the average blood glucose level: 28.3 × HbA1c – 43.9 ( 9 ). Our study used linear regression analysis to evaluate the correlation between fructosamine and mean glucose levels, and observed that for each increase of 1.0 µmol/L in fructosamine level there was a 0.5 mg/dL increase in mean glucose level, as evidenced in the proposed equation *Mean glucose level = (0.5157 x fructosamine level – 20.*

The coefficient of determination allowed the calculation of the estimated mean glucose value from the fructosamine value. The results of our study support a linear relationship between fructosamine and average glucose levels.

There is growing interest in the use of fructosamine in the screening and assessment of metabolic control in DM. Measurement of fructosamine levels has been proposed to improve the diagnosis and monitoring of DM in the face of the pitfalls that can occur with HbA1c measurement ( 10 ). Additionally, fructosamine measurement has been proposed as a predictor of DM risk ( 11 ). Thus, fructosamine is closely associated with DM risk, and the increase in fructosamine level can be a useful indicator of future DM risk, independent of blood glucose measurements.

The normal fructosamine value varies between 200-285 µmol/L when the serum albumin concentration level is 5 g/dL, and the reference values of the assay vary between 205-285 µmol/L ( 12 ). A study estimating the prevalence of DM using an oral glucose tolerance test (76 grams) and measurement of fructosamine levels, establishing as a cutoff value for the diagnosis of DM a fructosamine value of 310 µmol/L, has shown that the standard error rates of the estimated prevalence of DM ranged from 40% for a population of 200 individuals to 20% for a population of 2,000 individuals or more ( 13 ). The results of the present study show that our proposed equation allows the calculation of the average blood glucose level from fructosamine values in individuals with DM and the estimation of short-term glycemic control.

In conclusion, our study demonstrated a linear correlation between fructosamine levels and mean blood glucose levels, suggesting that fructosamine levels can be a proxy for the average glucose level in assessing metabolic control in patients with DM. Still, further studies are needed to validate the relationship between serum fructosamine levels and average blood glucose levels.
